# Notes on an Unusual Case of Hodgkin's Disease
1Read at a Meeting of the Bath and Bristol Branch of the British Medical Association on March 27th, 1912.


**Published:** 1912-06

**Authors:** E. Leonard Lees, F. H. Edgeworth


### NOTES ON AN UNUSUAL CASE OF HODGKIN'S DISEASE.[1]

BY

E. LEONARD LEES, M.D., and F. H. EDGEWORTH, M.D., D.Sc.

THE following case of Hodgkin's disease is perhaps worthy of being brought before your notice, as it showed the exceptional features of lymphoid growths in orbits, eyelids, temporal regions, and left mamma.

A man, aged 52, who had lived in Johannesburg for the previous twenty-three years, and who had not suffered from any illness, came under our care in April of last year. He had noticed four months before a swelling in the right side of the neck, and then swellings in successively the right axilla, left side of neck, left axilla, left groin, and right groin; also that the throat had swollen. Some loss of weight and failure of strength and energy had accompanied the development of these swellings.

On examination, it was found that the lymphatic glands in the above-mentioned regions were enlarged, discrete, painless,

[1] Read at a Meeting of the Bath and Bristol Branch of the British Medical Association on March 27th, 1912.

firm, and not adherent to the overlying skin. There were no symptoms or physical signs of enlargement of lymphatic glands in the chest and abdomen, nor any evidence of enlargement of the thymus gland. The liver was not enlarged; the lower pole of the spleen extended some two inches below the left costal margin. The tonsils and lymphatic nodules in the wall of the pharynx were enlarged, so that nasal breathing was difficult and the mouth consequently dry. Each temporal region was prominent, as if due to something bulging the temporal muscle outwards. Exophthalmos was doubtful; both eyelids were swollen, due to a deposit of firm tissue between the tarsal cartilages and the skin.

Examination of the blood gave the following results:—Number of red corpuscles 6,300,000, white corpuscles 2,000, per c.m. A differential count showed polymorphonuclears 52 per cent., large lymphocytes 7 per cent., small lymphocytes 27 per cent., eosinophils 14 per cent.

The administration of arsenic was immediately begun and persisted in. The dose given was liquor arsenicalis ♏ vii *ter die p.c.* Under this treatment the patient steadily improved, and by the end of July appeared almost well. The swellings in the eyelids and temporal regions were barely noticeable, the tonsils were of normal size, the lymphatic glands were palpable but of normal size. The spleen could not be felt until the patient was asked to take a deep breath, when it was found to descend so that it could be grasped and its upper edge felt.

The patient, who continued to take arsenic in smaller doses, remained well during the month of August and the first half of September, and then suddenly relapsed, so that by the end of the first week in October his condition was very like that present in the previous April. The following differences existed. The spleen was larger, extending down below the level of the umbilicus, and in addition to the swelling of the temporal regions and eyelids exophthalmos was now certainly present. The lymphatic glands and tonsils were as large as formerly. Examination of the blood showed 4,308,000 red corpuscles and 1,560 white corpuscles per c.m., hæmoglobin 70 per cent., colour index .81. A differential count of the white corpuscles gave polymorphonuclears 53 per cent., large lymphocytes 16 per cent., small lymphocytes 20 per cent., eosinophils 11 per cent.

From this time onwards the patient progressively grew worse. The liver began to enlarge, so that a month later it extended 2 in. below the right costal margin, and the spleen grew still larger. Fever, hitherto absent, came on, the temperature rising to 101° every afternoon from a normal one in the morning. The only further change, other than a steadily increasing weakness and rapidity of the pulse, was an enlargement of the left mammary gland, which eventually measured 1½ in. in diameter and ¾ in. in thickness. The right mammary gland did not enlarge. The patient died at the end of November, after an illness of eleven months' duration.

Permission to make a complete *post-mortem* examination was withheld, but we were permitted to excise a lymphatic gland from the left axilla. This gland was as large as a pigeon's egg with a slightly thickened capsule, and on section showed a slightly convex almost translucent appearance, with no caseation. Microscopical examination disclosed the existence of a delicate reticulum of varying mesh, enclosing lymphocytes, polymorphs, eosinophils, and uni- and multi-nuclear giant cells. No areas of necrosis were seen.

On reviewing the details of this case, it is clear that its features were similar to those usually seen and described in Hodgkin's disease—the characteristic enlargement of the lymphatic glands, beginning in one side of the neck and spreading from group to group, the enlargement of the spleen, the immense improvement under the influence of arsenic, the febrile relapse uninfluenced by this drug, the macroscopic and microscopic appearances of a lymphatic gland.

The condition of the blood was a little exceptional, in that a polycythemia was present in the early stages; later on the number of red corpuscles diminished somewhat, and an anæmia of secondary type developed. The number of leucocytes in Hodgkin's disease varies within rather wide limits, but diminution to the number found in this case is exceptional. A relative increase in the number of eosinophils is common, though not invariable; for instance, Pepper found an eosinophilia of from 8.8 to 14 per cent. in four out of twenty cases.

The extraordinary mobility of the spleen on resumption of its normal size in the month of July was probably due to a previous stretching of the phreno-splenic and posterior splenic ligaments.

The greatest interest, however, attaches to the presence of adenoid growths in the eyelids, orbits, temporal regions, and left mamma.

Exophthalmos and swelling of the eyelids have generally but not invariably occurred together, and then on both sides. They may also develop in diseases other than Hodgkin's. Parsons, in his *Pathology of the Eye,* groups such cases into lymphoma, leukæmia, pseudo-leukæmia, and doubtful. The associated features in the above recorded case definitely mark it out as one of lymphadenoma.

Swelling of the temporal regions and of the mammary gland must be still more exceptional in cases of lymphadenoma. We have not been able to find any record of their occurrence.

